# Optimization of Arabinoxylan Isolation from Rye Bran by Adapting Extraction Solvent and Use of Enzymes

**DOI:** 10.1111/1750-3841.13920

**Published:** 2017-09-29

**Authors:** Denisse Bender, Renata Nemeth, Michaela Wimmer, Sylvia Götschhofer, Matilde Biolchi, Kitti Török, Sandor Tömösközi, Stefano D'Amico, Regine Schoenlechner

**Affiliations:** ^1^ Dept. of Food Science and Technology BOKU‐ Univ. of Natural Resources and Life Sciences Muthgasse 18 1190 Vienna Austria; ^2^ Dept. of Applied Biotechnology and Food Science Budapest Univ. of Technology and Economics Müegyetem rkp.3 1111 Budapest Hungary; ^3^ Dept. of Food and Nutritional Sciences Univ. degli Studi di Milano Via Celoria 2 20133 Milano Italy

**Keywords:** arabinoxylan, chemical extraction, enzymatic extraction, rye bran, xylanases

## Abstract

Physicochemical and functional properties of arabinoxylans (AXs) can be significantly influenced by their isolation method. Finding balanced process conditions that allow optimal extraction yields while preserving AXs functionality is a challenge. The aim of this study was to determine the effect of different chemical solvents with neutral and alkaline pH on the intrinsic properties and extraction yield of AXs isolated from rye bran. Additionally, the application of xylanases and other cell wall degrading enzymes (Pentopan Mono BG, Deltazym XL‐VR, Viscoflow BG) to solubilize bound AXs was investigated. Results show that the use of Ca(OH)_2_ for isolation was superior to water and Na_2_CO_3_, as it selectively solubilized AXs and delivered isolates with a purity of up to 43.92% AX and a moderate ferulic acid (FA) content (209.35 ± 16.79 mg FA/100 g AX). Application of xylanases was further able to duplicate these achieved AX yields (7.50 to 9.85g AX/100 g bran). Additionally, isolates displayed highest ferulic acid contents (445.18 to 616.71 mg FA/100 g AX) and lowest impurities in comparison to chemical extracted AXs. Rheological characterization of the isolates showed a pronounced shear thinning behavior which fitted well to the power‐law model (*R* ^2^ > 0.989). Differences in pseudoplasticity of the isolates suggested that structural and chemical properties might have been responsible for this behavior.

## Introduction

The most common method to isolate arabinoxylans (AXs) from grains is by water extraction. Since most of the AXs found in the cell wall are embedded in a complex matrix with other cell wall components or with themselves, a limited amount of AXs are water soluble. Therefore, more severe treatments such as chemical, enzymatic or mechanically assisted treatments have been applied (Zhang and others 2014).

Alkaline solvents (for example, NaOH, KOH and Ca(OH)_2_) have efficiently solubilized AXs from cell wall materials, as they disrupt hydrogen and covalent bonds which loosen up the cell wall matrix. It has also been found that some divalent bases such as barium hydroxide, show a higher selectivity towards AXs as they interact with pentose sugars, which avoids co‐extraction of β‐glucan (Bergmans and others 1996). However, alkaline solvents may break down functional groups of AXs, reducing its functional properties. Mansberger and others (2014) isolated AXs from rye bran using low temperatures (20 and 30 °C) and different NaOH concentrations. Results showed that an increase in pH and temperature led to higher extraction yields of up to 2.5 g AX/100 g rye bran. Comparatively, Bender and others (2017) simplified the previous extraction process by adapting a broader temperature range (up to 70 °C) with different NaOH concentrations and could therefore promote a higher yield of 3.3 g AX/ 100 g bran. The study demonstrated that harsher extraction conditions contributed to the breakdown of bound FA, which partly decreased the isolate´s crosslinking ability. It showed a lower initial viscosity in solution when it was rheologically characterized in comparison to the isolates recovered at mildest extraction conditions.

A more gentle treatment for AX isolation is enzymatic extraction. Xylanases are able to solubilize AXs by hydrolyzing the β‐(1‐4)‐linkages between xylopyranoside residues in a random manner, causing partial solubilization of water insoluble AXs and depolymerization of water soluble AXs (Courtin and Delcour 2001). Most commonly, endo‐β‐(1, 4)‐xylanases from glycoside hydrolase (GH) family 10 and 11 have been applied for this purpose. Depending on the cleaving enzyme, AXs with different properties can be solubilized. Xylanases from GH11 families are characterized for their high substrate selectivity, high catalytic efficiency and preferentially cleave unsubstituted areas of the AX backbone chain. On the other hand, xylanases from GH10 family exhibit greater catalytic versatility, lower substrate specificity and have the ability to hydrolyze xylose linkages closer to the side chain residues (Motta and others 2013).

AXs can form covalently stabilized gels under oxidizing conditions by cross‐linking of AX chains through dimerization of FA substituents (Vinkx and Delcour 1996). This leads to a strong increase in viscosity in solution, as the molecular weight of the AXs increases. Since FA is mainly responsible for the cross‐linking behavior of AXs, the quantity of FA substituents and its distribution along the polymer chain can significantly influence their gelling properties (Dervilly‐Pinel and others 2001). A study made by Brenna and Cantarelly (1992), proved the key role of FA on the functionality of AXs in dough. Native AXs from rye and wheat flour greatly improved the viscoelastic properties of dough, whereas dephenolated AXs did not show any significant effect. Moreover, structural characteristics of AXs such as molecular weight and branching degree play also a significant role on their functionality and can be modified upon extraction.

Based on previous studies (Bender and others 2017; Mansberger and others 2014), the aim of this study was to further improve and optimize the arabinoxylan isolation procedure in order to obtain on the one hand sufficient yield but on the other hand also improve functional properties of the isolates. To pursue this aim different extraction solvents were investigated: water, sodium carbonate and calcium hydroxide; additionally xylanases were applied to release remaining bound arabinoxylans, which should result in an overall increased yield. All obtained isolates were characterized in regards to their chemical composition. Their rheological properties were preliminary evaluated in order to determine their ability to be used as baking improvers in gluten‐free bread.

## Materials and Methods

### Materials

Rye bran flour donated from Good Mills Austria GmbH (Schwechat, Austria) was used for AX extraction. Sodium carbonate and calcium hydroxide were bought from Sigma‐Aldrich Co. (Steinheim, Germany). For enzymatic treatment alpha‐amylase (Termamyl 120 L), amyloglucosidase (AMG 300 L), subtilisin (Alcalase 2.4 L), bacillolysin (Neutrase 0.8 L), endo‐1,4‐β‐xylanase (Pentopan mono BG) and endo‐1,3‐β‐glucanase (Viscoflow MG, a mixture of beta‐glucanases, xylanases, cellulases and alpha‐amylase) were purchased from Novozymes Ltd. (Bagsvaerd, Denmark). Xylanase from *Trichoderma longibrachiatum* (Deltazym VR‐XL) was donated from WeissBioTech GmbH (Ascheberg, Germany) and *Aspergillus niger* propyl‐endoprotease (AN‐PEP) was donated from DSM Food Specialties B.V. (Delft, The Netherlands). All used reagents were of analytical grade and purchased from Sigma‐Aldrich Co. (Steinheim, Germany).

### Extraction procedure with varied solvents (pilot scale) and by application of xylanases (lab scale)

**Figure 1:**
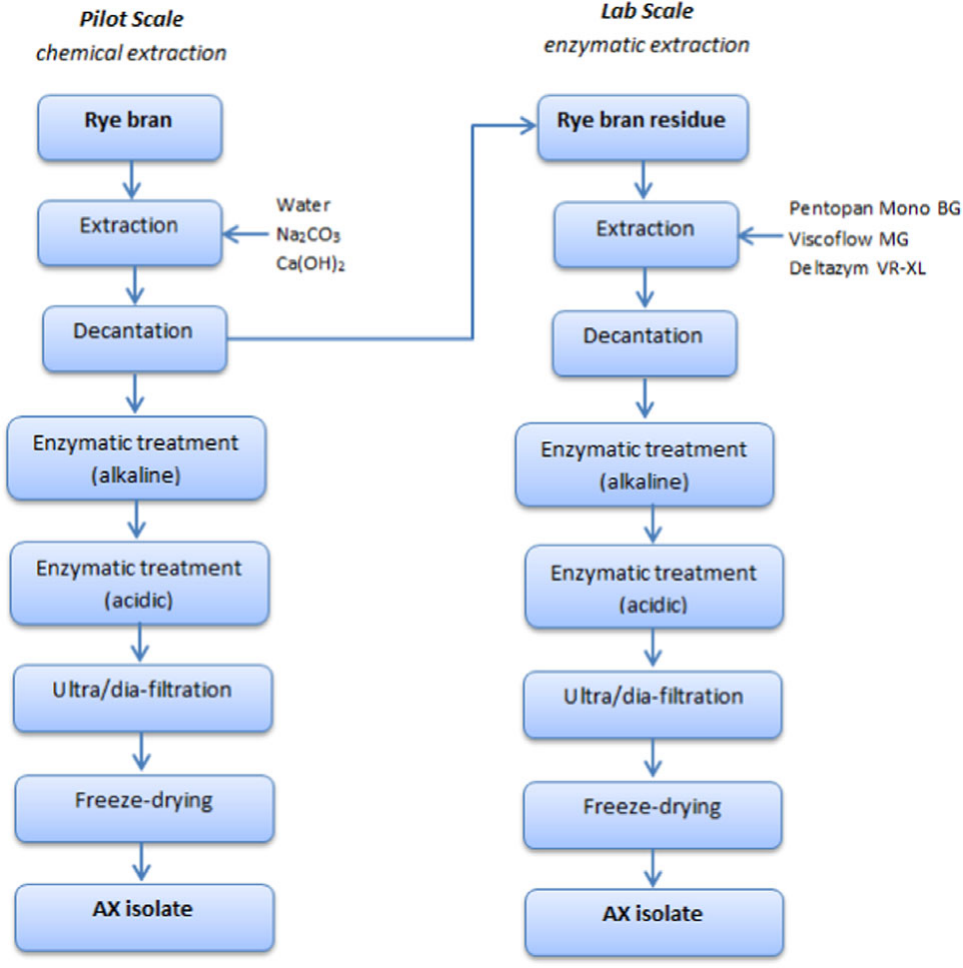
Chemical and enzymatic AX extraction from rye bran.

Figure 1 displays the flow chart for the extraction of AX by chemical solvents and by use of enzymes (selected xylanases).

For α‐amylase inactivation, the bran (4 kg) was dry heated at 130 °C for 90 min in an oven (Memmert GmbH & Co. KG, Schwabach, Germany). Sodium carbonate and calcium hydroxide were used as solvents at a concentration of 0.17 M (pH 10.18 and 12.18, respectively) and mixed with rye bran in a ratio of 1:10. For comparison, extraction with water was carried out. All extractions were performed at 65 °C for 100 min and constant agitation. Consequently, solids (rye bran residues) were removed using a horizontal centrifuge decanter (Sharples, Waldkraiburg, Germany) and pH was reduced to pH 6 with phosphoric acid. Enzymatic degradation of soluble starch (0.2 mL/L Thermamyl® and 0.2 mL/L AMG®) and protein (0.1 mL/L Neutrase®) at 55 °C for 3 h was performed. Then the pH was set to 8.5 with sodium hydroxide for further protease treatment (0.2 mL/L Alcalase® at 60 °C for 1.5 h). Furthermore, pH and temperature were reduced to 5.5 and 45 °C for gluten hydrolysis with AN‐PEP (0.145 mL/L) for 2 h. The obtained extract was preserved with 0.15 g/L of potassium sorbate. Enzymes were inactivated by heat treatment for 20 minutes at 95 °C; subsequently the extract was ultra/dia‐filtrated using a 8 kDa ceramic membrane (TAMI Industries, Hermsdorf, Germany) at 50 °C. The remaining solution was then freeze‐dried (FreeZone 6, Fa. Labconco, Kansas City, Mo., U.S.A.).

In order to improve AX extraction yield, the rye bran residues separated during the pilot scale extraction with water were vacuum‐dried at 50 °C for 12 h (Series VD, Fa. Binder GmbH, Germany). Xylanase extraction was performed by dissolving 200 g of rye bran residue in 2 L of distilled water. An initial concentration of 600 U of Viscoflow MG was selected for extraction. Since low arabinoxylan content was recovered at this step, dosage in further trials was increased to 3000 U of Pentopan Mono BG and 3000 U of Deltazym VR‐XL for further extractions. Each mixture was heated up with constant agitation to 50 °C, kept for 90 min and then solids were removed by filtration (Whatmann® filter paper, Buckinghamshire, U.K.). Subsequent enzymatic degradation of starch and protein was carried out following the procedure described above. Since gluten detoxification was not achieved in chemical extraction trials, AN‐PEP addition was raised to 0.2 mL/L. The extract was ultra/dia‐filtrated, recirculating the extraction solution 10 times with a 5, 10, and 30 kDa ceramic membrane from Sartorius AG (Göttingen, Germany) and freeze‐dried (FreeZone 6, Fa. Labconco).

All extractions were carried out in duplicate.

### Analytical methods

To determine the influence of different extraction procedures on their chemical composition, AX isolates were analyzed by different methods. Dry matter was determined according to ICC‐standard method 110/1 (110/1 1976). Total starch was performed according to the standard method of AACC No. 76‐13.01 (AACC 2000) (Megazyme test kit, Megazyme International Ireland Ltd., Wicklow, Ireland). Soluble and insoluble dietary fiber (SDF and IDF) was determined following the standard method of AACC No. 32‐07 (AACC 2000) (Megazyme test kit, Megazyme International Ireland Ltd., Wicklow, Ireland). Protein content was performed according to the ICC standard 167 (Dumas) (167 2000). Bound phenolic compounds of rye bran and AX isolates were analyzed as reported by Mattila and others (2005). The measurement of monosaccharide composition was carried out following the procedure of Gebruers and others (2009), with slight modification. Samples were hydrolyzed with TFA and reduced with NaBH_4_ in the presence of ammonia. Alditol acetate derivatives were formed using acetic anhydride. After derivatization monosaccharides were determined by using Perkin Elmer Clarus 500 gas chromatograph. Elite‐17 capillary column (60 m x 0.5 mm I.D. and film thickness of 1 μm, Perkin Elmer, Waltham, Mass., U.S.A.) with a methyl‐polysiloxane phase was used. The carrier gas was helium with initial column flow of 1 mL/min. The injector temperature was 300 °C with 1 μL injection volume. Monosaccharides were detected with flame ionization detector (FID) on 300 °C. Detector gases were air (450 mL/min) and hydrogen (45 mL/min). All analyses were performed in triplicate, except for monosaccharide composition and gluten content, which were performed in duplicate. The AX content was calculated as the sum of arabinose and xylose fractions.

### Gluten quantification

Gluten content of AX isolates was quantified using a competitive Enzyme Linked Immunosorbent Assay (ELISA) using the R5 antibody (Ridascreen® Gliadin competitive, R‐Biopharm, Darmstadt, Germany). Gliadin concentrations were calculated using Microplate Manager 6.0 (Bio‐Rad, Tokyo, Japan) computer software with a Logit‐Log fit and converted into gluten concentration, by multiplying the gliadin concentration by a factor of 2 (Codex Alimentarius 2008).

### Rheological measurements

Rheological characterization of AX isolates was performed using a Kinexus Rheometer pro+ (KNX 2001, Malvern Instruments GmbH, Herrenberg, Germany) at 25 °C. 100 mg of each sample was diluted in 10 mL distilled water and immediately poured on a cone‐plate geometry (CP1/60). Flow curves were obtained under continuous shearing over a shear rate range from 0.01 to 10 s^−1^. Measurements were performed at least in triplicate and rheological parameters were evaluated using the manufacturer's supplied computer software (rSpace for Kinexus, Malvern Instruments GmbH, Herrenberg, Germany). Rheological behavior of AXs was described by fitting the experimental data to a power law model.

$$\eta =K{\dot{\gamma }}^{n-1}$$

where ɳ is apparent viscosity (Pa‐s), K is the consistency coefficient (Pa‐s^n^), $\dot{\gamma }$ is the shear rate (s^−1^) and n is flow behavior index.

### Data analysis

Statistical analyses were performed using STATGRAPHICS Centurion XVII, version 17.1.04 (Statpoint Technologies, Inc., Warrenton, Va., U.S.A.). Results of all analysis are expressed as mean ± standard deviation of at least 3 replicates of each sample. One‐way ANOVA (analysis of variance with α = 0.05) and Fishers least significance tests were used to determine statistical significant differences between the samples. Significant differences were indicated by different letters in the rows when p‐value was lower or equal to 0.05.

## Results and Discussion

As mentioned before, extraction yield and macromolecular characteristics of AXs can vary according to the isolation method, thus the influence of different solvents or enzymes on the extractability and properties of AXs was evaluated. The results for the detailed characterization of the obtained AXs are provided in Table 1 and 2, in comparison to the chemical composition of the original rye bran.

**Table 1:** Influence of selected solvents on the chemical composition of AX isolates extracted at 65°C and 0.17M in comparison to the raw material

		Protein	Starch	β‐Glucan	AX content a		Bound FA	Bound FA	Yield	Gluten
Extraction		(g/100 g)	(g/100 g)	(g/100 g)	(g/100 g), n = 2	A/X ratio	(mg/100 g), n = 3	(mg/100 g AX), n = 3	(g AX /100 g rye bran)	(ppm), n = 2
Rye bran		14.94 ± 0.03	19.03 ± 1.04	4.05 ± 0.55	33.36 ± 0.94	0.38 ± 0.00	223.01 ± 13.01	668.57 ± 39.01		N.D. b
*Solvent*	*pH* c									
Water	6.43	10.99 ± 0.86a	2.22 ± 0.18c	6.40 ± 0.22b	38.57 ± 0.09a	0.48 ± 0.01a	137.30 ± 14.41b	356.01 ± 37.35b	2.92	99.25c
Na_2_CO_3_	10.2	12.46 ± 0.17b	0.98 ± 0.05b	7.59 ± 0.07c	41.80 ± 0.18b	0.53 ± 0.00a	108.32 ± 10.23a	259.15 ± 24.46a	3.85	33.11b
Ca(OH)_2_	12.2	14.04 ± 0.82c	0.08 ± 0.01a	0.43 ± 0.04a	43.92 ± 0.42b	0.59 ± 0.01b	94.25 ± 7.56a	209.35 ± 16.79a	3.81	6.80a

Mean value of triplicate determinations ± standard deviation. Values associated with different lower case letters denote significant differences (*P* < 0.05).

^a^AX was calculated as the sum of arabinose and xylose fractions.

^b^N.D.: not determined.

^c^pH of solvent used at a concentration of 0.17 M.

**Table 2:** Influence of the selection of xylanases on the chemical composition of AX isolates extracted at 50 °C

	Protein	Starch	β‐Glucan	AX content a		Bound Ferulic acid	Bound Ferulic acid	Yield	Gluten
Extraction	(g/100 g)	(g/100 g)	(g/100 g)	(g/100 g)	A/X ratio	(mg/100 g), n = 3	(mg/100 g AX), n = 3	(g AX /100 g rye bran)	(ppm), n = 2
Rye bran residue	9.10 ± 0.33	15.83 ± 0.56	8.99 ± 0.28	62.77 ± 0.18	0.38 ± 0.00	451.77 ± 10.56	719.73 ± 16.82		N.D. b
Xylanases									
Pentopan mono BG	4.13 ± 0.06b	0.27 ± 0.07	0.36 ± 0.11a	67.10 ± 6.86c	0.25 ± 0.00b	337.39 ± 41.30b	504.92 ± 43.77b	8.81	11.47
Viscoflow MG	4.25 ± 0.10b	0.25 ± 0.08	1.58 ± 0.23b	40.01 ± 1.39a	0.28 ± 0.00c	172.68 ± 11.72a	445.18 ± 33.00a	7.50	4.28
Deltazym VR‐XL	3.18 ± 0.23a	0.27 ± 0.06	0.39 ± 0.03a	53.41 ± 5.02b	0.23 ± 0.00a	305.22 ± 36.95b	616.71 ± 61.96c	9.85	12.45

Mean value of triplicate determinations ± standard deviation. Values associated with different lower case letters denote significant differences (*P* < 0.05).

^a^AX was calculated as the sum of arabinose and xylose fractions.

^b^N.D.: not determined.

### Influence of selected solvents on extraction of arabinoxylan

Rye bran exhibited a moderate amount of protein and starch (see Table 1), but a high amount of dietary fiber, which was additionally measured (not shown in Table 1). Most of the fiber present was insoluble (43.56 ± 2.49%) and only a small fraction was found to be soluble (6.57 ± 0.79%). It is known that most of the dietary fiber in rye is constituted by AXs, which are mostly bound to other cell wall components.

Regarding the AX isolates, significant differences in the chemical composition could be observed, which varied depending on the selected solvent (see Table 1). Overall, low starch content and moderate amounts of protein were co‐extracted after the isolation process. Greatest protein solubility of 14.04 ± 0.82% was achieved when using Ca(OH)_2_, which decreased the purity of the isolate. As seen in other investigations (Lim and others 1999; Mansberger and others 2014; Wang and others 2014), alkaline solvents cause disruption of hydrogen bonds and increase the net charge of proteins, which increased the solubility of these impurities. Complete hydrolysis of starch molecules was achieved in the isolates treated with Ca(OH)_2_, slightly more was found in the isolates obtained by Na_2_CO_3_ extraction. These results might be explained by a higher degree of depolymerization, which is favored at alkaline pH. Hydrolyzed dextrins could then be removed easily by ultra/dia‐filtration. Co‐extraction of β‐glucan hardly occurred when using Ca(OH)_2_, in contrast to the other investigated solvents . Prior studies confirmed that bivalent cations might be responsible for the lower extractability of (1‐3, 1‐4)‐β‐glucans and have been used to selectively isolate AXs. A reduced solubility of β‐glucan has been reported in saturated Ca(OH)_2_ solutions (0.01 M) in earlier studies (Verbruggen and others 1995; Bergmans and others 1996).

The selection of solvent also influenced the AX content, solubilizing significantly greater amounts at a higher pH. Additionally, arabinose to xylose (A/X) ratio revealed an intermediate branching degree in all isolates, being higher when using Ca(OH)_2._ It has also been reported that low concentrations of alkaline solvents were able to extract AXs with higher substitution degrees (Bergmans and others 1996). Alkaline extractions (Na_2_CO_3_ and Ca(OH)_2_) resulted in higher yields of isolated AXs than water extraction, which was even higher than several reported studies (Hartmann and others 2005; Buksa and others 2013; Mansberger and others 2014).

Similarly, statistical analyses of data (Table 1) suggest that there was a significant influence (*P* < 0.05) of the solvent on FA and gluten content. Since the crosslinking behavior highly relies on the ability of FA to connect one AX to another AX‐chain or even to other structural polymers (for example, lignin, cellulose, proteins) when it is oxidized (Vinkx and Delcour 1996), isolates with high FA content are favored. Additionally, the FA content of AXs has been positively correlated with molecular weight (Hartmann and others 2005), which in turn would most likely improve the viscoelastic properties when added to a dough. Highest amount of bound FA was found when using water and lowest in the isolates extracted with Na_2_CO_3_ and Ca(OH)_2_. Zhou and others (2010) suggested that alkaline media may break down the ester bond between FA side chain and AXs, releasing the FA, which would explain the lower content found when using Na_2_CO_3_ and Ca(OH)_2_ in comparison to water. With reference to the gluten content, highest contamination was found in the isolate extracted with water, which along with Na_2_CO_3_ exceeded the threshold for gluten‐free products (20 ppm). Only the Ca(OH)_2_ extraction was able to meet this requirement. It is likely that the high alkalinity of Ca(OH)_2_ led to a higher solubilization of protein and gluten, but simultaneously increased the gluten hydrolysis, which was efficiently removed during the purification process. The high extraction temperature had a significant influence as well. Bender and others (2017) showed that higher extraction temperatures (up to 70 °C) led to higher co‐extraction of proteins under alkaline conditions, but lower gluten content.

Overall, results showed that the selection of solvents significantly influenced chemical composition of AX isolates. Best AX extraction was shown by Ca(OH)_2,_ which not only extracted selectively a higher amount of AXs in comparison to water, but also displayed a moderate FA content and fulfilled the specifications for application in gluten‐free products. However, compared with the outgoing rye bran, still much of the bound AX could not be extracted. Thus, the aim of the next trials was to increase the yield of AX isolation by application of enzymes in order to release bound AXs from the bran (see Figure 1).

### Influence of enzymatic treatment on lab scale AX‐extraction

The use of xylanases has shown great potential to increase AX extraction yield, targeting insoluble AX which still remain in the rye bran matrix (Zhang and others 2014).

AX isolates were characterized by means of their chemical composition, as described in Table 2 and compared with the rye bran residue from which it was previously extracted. Considering that most of the protein and starch content was already solubilized during the first extraction, co‐extraction of impurities was rather limited. Protein and starch content of isolates were below 4.13 and 0.27%, respectively. Moreover, only small amounts of β‐glucan were co‐extracted during isolation, being significantly higher when using Viscoflow MG, which is mainly comprised of an endo 1,3‐β‐glucanase. These lower impurities resulted in higher AX contents in these enzyme extracted isolates, which reached up to 67.10% by use of Pentopan mono BG. The high catalytic efficiency of Pentopan mono BG has already been reported earlier (Falck and others 2014). Its performance might be explained by the high selectivity of its GH11 xylanase for insoluble substrates. Another explanation is its increased ability to penetrate the cell wall network (Motta and others 2013). As for Deltazym VR‐XL, high AX cleavage was also possible. It is likely that the xylanase present in this enzyme preparation might also belong to the GH11 family, as it showed similar release properties and low A/X ratios. Since *Trichoderma longibranchiatum* can produce more than one kind of xylanase (Royer and Nakas 1991) and product specification of Deltazym VR‐XL was not provided, only speculation in this matter is possible. Lowest AX content was observed when using Viscoflow MG, which is related to its lower substrate specificity. Another possibility for the inferior AX solubilization would be the lower dosage of the enzyme preparation used (600 U in comparison to 3000 U for the other enzymes).

In general, a minor substitution degree (0.23 to 0.28) was observed in contrast to the chemical extracted AXs (0.48 to 0.59). It has been reported that xylanases from the family GH11 cannot fully hydrolyze AXs from highly substituted parts of the bran because of its narrow substrate‐binding cleft, favoring hydrolysis from less substituted segments (Falck and others 2014).

Overall, enzymatic extraction was able to duplicate the extraction yield compared with chemical isolation. Since xylanases targeted mainly dietary fiber components, without cleaving almost any impurity, extraction of AXs was more efficient than water and alkaline extraction. FA content of AX isolates was superior to chemical extracted AXs. A similar outcome has been reported by Zhou and others (2010), where Pentopan Mono BG showed significantly higher amounts of bound FA in comparison to the alkaline extracted isolates. As for gluten detoxification, all enzymatic extractions were able to successfully eliminate CD toxic gluten contamination, in contrast to chemical extractions (Table 1). Considering that the extraction was performed using the rye bran residue from the first extraction step (Figure 1), part of the original proteins was already extracted off and thus a lower solubilization of proteins and therefore gluten was possible. This variable combined with a higher dosage of AN‐PEP, could effectively degrade the CD‐toxic epitopes, providing gluten‐free isolates.

Xylanase treatment showed to be an advantageous method for AX isolation, since it produced the highest amounts of isolates with highest purity and low molecular damage (that is, ferulic acid content). It provides an environmentally friendly alternative to traditional (alkaline) extraction methods.

If these 2 processes are combined as described in Figure 1, the first step the extraction of the soluble AX with an alkaline solution (for example, CaOH_2_) and the second step applying an enzymatic extraction targeting only the remaining bound AXs in the rye bran residue, the final yield and functionality (that is, FA content) of isolated AXs can be increased immensely. Important is that these 2 extraction steps are performed separately. In other words, the decantation step after the alkaline extraction seemed to play a significant role during the extraction. This investigation also evaluated the one‐step chemical and enzymatic extraction, by eliminating the decantation of the rye bran residue and adjusting the pH in‐between the 2 extractions (detailed data not shown). Surprisingly, the one‐step extraction led to a lower AX yield (2.01 g AX/100 g rye bran). It is believed that during the xylanase extraction, xylanases depolymerized the solubilized AXs from the first extraction step to such a degree, that they were filtered out during purification.

### Rheological characterization of AX isolates

**Figure 2:**
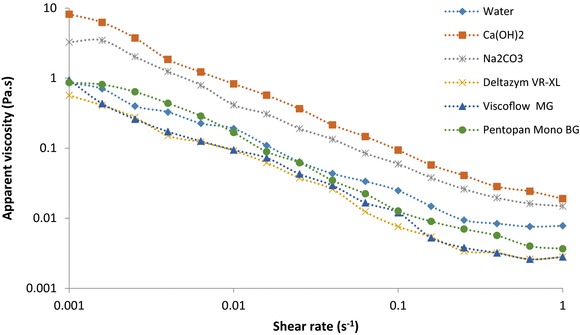
Shear rate dependence of viscosity of chemically and enzymatically isolated AXs in 1% solutions.

Steady‐state shear properties of AX isolates were monitored over a specific range of shear rates and steady‐state flow curves of 1% AX solutions are shown in Figure 2. As this shows, at lower shear rates a region corresponding to the Newtonian plateau can be observed, followed by a region of decreasing viscosity as shear rate increased. Such flow patterns are typical for shear‐thinning materials (Izydorczyk and others 1991). Overall, all samples showed a pseudo‐plastic behavior, which can also be confirmed by the flow behavior indices (n < 1) as seen in Table 3. AX isolated with Ca(OH)_2_ and Na_2_CO_3_ displayed highest viscosities. Poorest viscosities could be seen in isolates extracted with Viscoflow MG and Deltazym VR‐XL.

**Table 3:** Power law model parameters of 1% AX solutions

	Model parameters		
AX‐ Isolate	K (Pa‐s^n^)	n	R^2^
Water	0.0039	0.205	0.991
Ca(OH)_2_	0.0116	0.059	0.995
Na_2_CO_3_	0.0077	0.089	0.993
Pentopan Mono BG	0.0019	0.045	0.990
Deltazym VR‐XL	0.0012	0.101	0.989
Viscoflow MG	0.0014	0.110	0.991

Within the linear region, flow curves of AX isolates were fitted to the Power‐Law model in order to describe the apparent viscosity as a function of shear rate (Table 3). All data fitted well within the model, as R^2^ values remained above 0.989. K and n parameters of Power law model depended on the type of extraction. Higher flow consistency indices (K) were shown by chemical extracted AXs, being most pronounced by Ca(OH)_2_. On the other hand, no defined tendency was recognized in case of the flow behavior index (n). Lowest n, and therefore highest pseudoplastic behaviors were displayed by the isolates extracted with Pentopan Mono BG and Ca(OH)_2_. Water isolate was found to be the least shear thinning. It is suggested that these outcomes were a consequence of a combined effect of factors. Differences in molecular weight of AX isolates might have had an impact on their flow behavior as seen by Kale and others (2015). In like manner, Izydorczyk and Biliaderis (1995) observed that the viscosity and shear thinning behavior not only depended on the molecular size of the polymer, but also on their asymmetrical conformation, since arabinose residues could stiffen the AX molecule along the chain. These investigators proposed that the specific arrangement of arabinose residues along the backbone chain could also be an important factor as intermolecular alignment between unsubstituted AX molecules might occur, which in turn would lead to aggregation of AX isolates. In addition, polymeric impurities remaining in the isolates, such as starch or proteins could also have an influence on the rheological properties. Another aspect that should be contemplated is the cross‐linking ability of the AX isolates. Buksa and others (2014) studied shear properties of cross‐linked and hydrolyzed AX isolates. Cross‐linked AX revealed highest apparent viscosity and lowest flow behavior index. Since cross‐linked molecules have a more complex structure and higher molecular mass, their behavior would differ most from Newtonian fluids.

To further define the functionality of AX isolates and verify its application in GF products, the next stage of this study will investigate the application of the produced AX isolates in simplified GF dough matrices.

## Conclusion

This study could successfully evaluate the influence of the selected isolation methods on the AX extraction from rye bran. Amongst these processes, additional enzymatic extraction has proven to increase AX yield, reduce impurities and preserve its functionality (that is, FA content). Moreover, this isolation procedure has shown superior economic feasibility and lower environmental impact than traditional (alkaline) extraction methods.

Also high purity reaching up to 67.10% of AXs in the isolates was achieved, considering that only the use of expensive alcoholic precipitation has so far been able to reach such purification yields. Characterization of the AX isolates was not fully able to provide sufficient information to understand the influence of extraction method on the rheological behavior of AXs. Therefore, gelling properties and functionality of AX isolates in GF simplified dough systems should be characterized next.

## Author Contributions

D. Bender performed the statistical analysis of the data and drafted the manuscript. R. Nemeth carried out the rheological characterization of all AX isolates, M. Wimmer carried out enzymatic AX extraction and characterization of the isolates, Sylvia Götschhofer performed pilot scale extractions and characterization of the AX isolates, Matilde Biolchi participated in the AX purification process, S. Tömösközi contributed the design and coordination of the study, K. Török performed the monosaccharide composition analysis and gluten quantification, R. Schoenlechner participated in the design of the study and helped to draft the manuscript, S. D'Amico contributed the design of the study, the interpretation of data and performed the critical revision of the manuscript. All authors read and approved the final manuscript.
